# Strategies for Exploiting Milk Protein Properties in Making Films and Coatings for Food Packaging: A Review

**DOI:** 10.3390/foods12061271

**Published:** 2023-03-16

**Authors:** Stefano Gerna, Paolo D’Incecco, Sara Limbo, Marta Sindaco, Luisa Pellegrino

**Affiliations:** Department of Food, Environmental and Nutritional Sciences (DeFENS), Università degli Studi di Milano, Via G. Celoria 2, 20133 Milan, Italy

**Keywords:** milk proteins, food packaging, coatings, biopolymers, plasticizers, water vapor permeability, tensile strength, bioplastics

## Abstract

Biopolymers of different natures (carbohydrates, proteins, etc.) recovered from by-products of industrial processes are increasingly being studied to obtain biomaterials as alternatives to conventional plastics, thus contributing to the implementation of a circular economy. The food industry generates huge amounts of by-products and waste, including unsold food products that reach the end of their shelf life and are no longer usable in the food chain. Milk proteins can be easily separated from dairy waste and adapted into effective bio-based polymeric materials. Firstly, this review describes the relevant properties of milk proteins and the approaches to modifying them for subsequent use. Then, we provide an overview of recent studies on the development of films and coatings based on milk proteins and, where available, their applications in food packaging. Comparisons among published studies were made based on the formulation as well as production conditions and technologies. The role of different additives and modifiers tested for the performances of films and coatings, such as water vapor permeability, tensile strength, and elongation at break, were reviewed. This review also outlines the limitations of milk-protein-based materials, such as moisture sensitivity and brittleness. Overall, milk proteins hold great potential as a sustainable alternative to petroleum-based polymers. However, their use in food packaging materials at an industrial level remains problematic.

## 1. Introduction

Dairy industries operate worldwide and produce many different food products, such as drinking milk, cheese, butter, fermented milk, and milk powders, where milk solids are recovered with different efficiency. Around 4–11 million tons of dairy waste is disposed of every year, and because of its high organic content, it represents a real hazard for the environment [1]. In addition, milk and dairy products include fresh products with an extremely short (1–2 weeks) shelf life. These products create further waste if they surpass their expiry date without being consumed. A practical example is pasteurized milk bottled in clear PET (polyethylene terephthalate) bottles and displayed in shops and supermarkets. Limbo et al. [2] emphasize that, depending on refrigeration conditions, the shelf life of this type of milk can be somewhat extended; however, this extension could cause decay of its nutritional and sensory properties due to light exposure. Expired milk quickly coagulates, sours, and spoils; therefore, it is often disposed of without recycling and taking advantage of its high-quality nutrients. Expired milk can be alternatively used for animal feed or soil fertilization.

The biotechnological valorization of whey has attracted great interest from researchers, while the management of expired milk or, more generally, dairy waste has received scarce attention in the literature until very recently. The use of dairy waste as a substrate in microbial fermentation reactors for producing value-added compounds [3], for the extraction of selected components by green processes [4], and as a conductive material for batteries [5] are among the recently proposed strategies. However, transferring these novel approaches to technological processes that can be used in industrial production remains problematic. Dairy waste differs in its composition and the characteristics of individual components; therefore, its reuse implies that targeted technologies are developed. Often pre-treatments are necessary steps in order to stabilize the product, remove contaminants, or simply concentrate targeted components, increasing the efficiency of the subsequent process. Components isolated from dairy waste or expired dairy products are normally sanitized and turned into powders to be used as new raw materials. The purity grade, in terms of residual microbiological and chemical contaminants, of these raw materials will define their suitability for further uses. As for any raw material or ingredients, safety and quality characteristics shall comply with the legal requirements provided for specific usages, either in food or non-food areas. Thus, the commercial valorization of the obtained derivatives will depend on both their origin and final destination. In principle, these ideal achievements help to mitigate environmental impacts while creating new employment opportunities within the framework of a sustainable industrialization.

**Figure 1:**
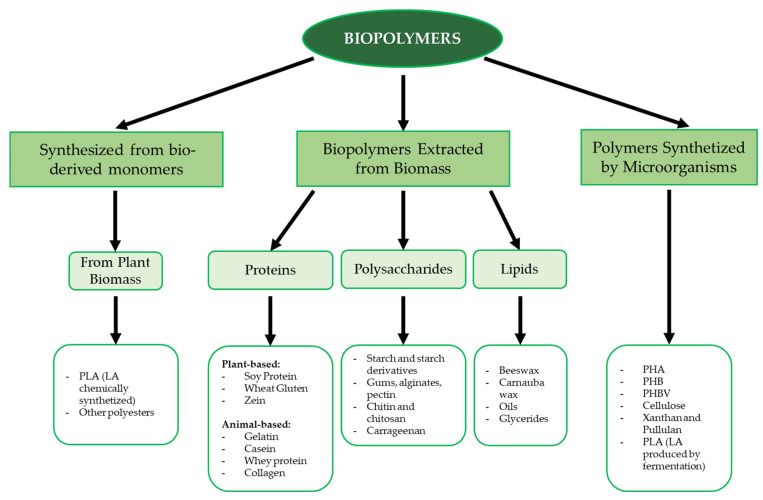
Schematic description of the main biopolymer classes. Abbreviations: PLA (polylactic acid), PHA (polyhydroxy alkanoate), PHB (polyhydroxy butyrate), PHBV (polyhydroxy butyrate-valerate).

The packaging sector also has an impact on the environment. Food products are generally packed after processing with the aim of both preventing microbial spoilage and preserving their sensory and quality properties during shelf life. The European Union policy on the sustainability of the whole food chain [6] and the “zero waste” goal of the Food and Agriculture Organization of the United Nations (FAO) [7] have encouraged food packaging manufacturers to find alternative, green packaging solutions; therefore, researchers are studying materials that can replace conventional plastic. Above all, the production of biodegradable packaging materials based on natural biopolymers is being prioritized, with these biopolymers being obtained from renewable sources or by-products of different origins [8,9]. Biopolymers derived from by-products or waste are already available and sometimes underutilized resources, and their use reduces disposal costs [10]. Biopolymers such as proteins, polysaccharides and lipids, with the aid of some additives, appear to be promising raw materials [11,12]. Biopolymers can be obtained from a variety of animal or plant sources; some can be chemically synthesized from bio-based monomers (e.g., polylactic acid) or synthesized by microorganisms (e.g., cellulose, polyhydroxyalkanoates, xanthan) [13]. An overview of the main biopolymer classes based on their origin and chemical nature is shown in Figure 1.

Among polysaccharides, the most common biopolymers include starch, cellulose, alginic and hyaluronic acid, dextran, chitosan, and chitin [14]. Due to their widespread availability, low processing costs, and wide range of properties and compositions, polysaccharide polymers are commonly used for various biological applications, such as food packaging, tissue engineering, and bioplastic formation [14,15]. With respect to proteins, the most used biopolymers are collagen, gelatin, soy protein, and milk proteins due to their biodegradable and biocompatible properties. This class of biopolymers also has excellent mechanical strength, heat resistance, and water resistance, making it suitable for storage films, packaging films, and heat-processed products [16]. Aliphatic polyesters are another class of polymeric macromolecules, with polylactic acid and polyhydroxy butyrate being the most significant materials.

Currently, multiple advantages, including high accessibility and biodegradability, widespread natural sources, mechanical properties, and the controlled release of additives and bioactive compounds, have sparked broader investigations on protein biopolymers and related applications compared with other biopolymers [17]. This review focuses on the main features of milk-protein-based films and coatings and discusses the available strategies used to improve their technological properties. The native properties of milk proteins are preliminarily presented in order to provide the reader with the correct background knowledge. Furthermore, the main strategies to modify these properties to improve milk protein performances are discussed. Finally, different uses and applications of milk-protein-based films and coatings are considered, depending on their formulations and additives.

## 2. Milk Proteins: Native Structures and Properties

Milk protein fractions include numerous proteins. Caseins are the most abundant (around 75–78%), being a class of phosphorylated proteins; the remaining 22–25% is represented by whey proteins, which are broadly defined as “soluble proteins”, and minor components such as proteose peptones. The relevant physico-chemical characteristics of milk proteins are described below and summarized in Table 1.

### 2.1. Caseins: Characteristics and Properties

Casein is defined as the protein fraction present in milk that precipitates at a pH of 4.6 at 20 °C [18]. Casein includes four principal components, αs1, αs2, β and κ, representing nearly 40, 10, 35, and 15% of the whole fraction, respectively, and with a molecular weight ranging from 20 to 25 kDa [19]. The primary structure of casein is characterized by a high content of proline, which interrupts the α-helices and β-sheet secondary structures, giving a disordered structure to the molecule. The tertiary structure is also loose and unexpressed. Consequently, caseins have a flexible structure with an open shape that can adapt to the surrounding environment. These features make them susceptible to proteolysis but resistant to heat treatments. The quaternary structure is called a micelle, which originates from the aggregation of hundreds of submicelles bound to each other by calcium phosphate clusters (6–8% by weight). The most recent studies on the structure of casein micelles depict them as highly hydrated sponge-like colloidal particles. Huppertz et al. [20] reported that water binds to 1 g casein in different ways: around 0.5 g of water directly binds to the protein, 1 g is associated with the glycosylated end of κ-casein, and 1.8 g occupies the pores of the micelles for a total hydration of ~3.3 g of water for 1 g of casein. Such a strong hydration occurs because casein micelles have a net negative charge in milk at a pH of 6.6–6.7. When milk is acidified to a pH of approximately 4.6, the micelles become more protonated, the calcium phosphate progressively solubilizes, and the casein molecules bind in small aggregates of insoluble acid casein.

Individual casein fractions display rather characteristic features, mostly depending on their amino acid composition and conformational flexibility. Caseins easily interact with multiple target molecules and thus are biologically fundamental in binding to surfaces and organizing in macroscopic networks [21]. The αs1-casein has a molecular weight of 23.6 kDa and a disordered “random coil” structure due to its high content (8.4%) of proline [18]. Differently, despite its similar molecular weight (25.2–25.4 kDa), the αs2-casein molecule can be divided into five distinct regions: two regions (residues 1–41 and 42–80) represent the typical casein phosphorylated regions with low hydrophobicity and high charge; a third region (residues 81–125) form a slightly positively charged region with high hydrophobicity; a fourth region (residues 126–170) with a negative charge and low phosphate content; finally, the last region (residues 171–207) has a high positive charge and high hydrophobicity [19]. β-casein has a molecular weight of 24.0 kDa and a random tangle shape with many β-turn structures. β-casein is the most hydrophobic among caseins, with a negatively charged hydrophilic N-terminal region formed by 40 amino acids residues. Its hydrophobicity increases in the C-end region. These properties of β-casein, unlike other unstructured disordered proteins, determine its ability to self-assemble into micelles under physiological conditions and even in acidic conditions. The hydrophobic part of the β-casein molecule is inside the micelle, while the hydrophilic part bearing the phosphorylation centers (mainly Ser-P residues) is located outside [21]. The κ-casein is the smallest of caseins (molecular weight of 19.0 kDa); it is a glycoprotein that contains about 5% of carbohydrates and two cysteine residues. In fact, the C-terminal part, named glycomacropeptide, is hydrophilic due to the presence of a glucidic moiety. The κ-casein is found on the external part of the micelle in a heterogeneous way, described as a brush layer [18]. It was also demonstrated that κ-casein does not have a stable three-dimensional structure, and this allows for changes in the conformation at different pH and temperature [22].

### 2.2. Whey Proteins

The proteins found in whey after either isoelectric precipitation or enzyme coagulation of caseins represent around 17% of protein substances in milk. Whey proteins include β-lactoglobulin (βLg), α-lactalbumin (α-La), bovine serum albumin (BSA), immunoglobulins (Igs), lactoferrin (Lf), lactoperoxidase (LP), and other indigenous enzymes [23,24]. The proteose peptones (PP) are also considered part of the whey protein fraction.

The β-Lg is the most abundant (51%) whey protein, with a molecular weight ranging from 18.20 to 18.36 kDa [25,26]. This protein exists as a dimer of two identical subunits, where each monomer entails one sulfhydryl group and two disulfide bonds. β-Lg is largely insoluble in distilled water, and salt dramatically increases its solubility. In this regard, the unusual solubility is the result of a strong salt binding due to the unique distribution of surface charges, and thus dipoles, at a neutral pH [25]. Thermal denaturation of β-Lg occurs at 70–75 °C, whereas aggregates form at 78–82 °C. The precipitation of β-Lg occurs before the precipitation of α-La due to lower heat resistance. The α-La, representing 22% of the whey protein fraction, is a small globular protein formed by a single polypeptide chain with a molecular mass of around 14 kDa. In the polypeptide chain, there are eight cysteine residues that form four disulfide bonds and thus no free cysteine sulfhydryl groups [26]. The α-La is a calcium-regulating protein and, more in general, can act as a metal carrier. The presence of a Ca^2+^ ion inside the molecule stabilizes its conformation; hence, removing calcium increases sensitivity to heat denaturation. The BSA represents 7–8% of the whey protein fraction and has a molecular mass of around 66 kDa [23]. An important property of BSA is its ability to bind in a reversible way to multiple ligands; therefore, this protein can be used as a carrier for fatty acids and other lipids [27]. The Igs include three main classes: IgG, IgA and IgM, with IgG including IgG1 and IgG2. They are the largest whey proteins with a molecular mass of up to around 180 kDa, and thus, they are highly susceptible to destabilization and precipitation.

## 3. Approaches and Methodologies for Modifying the Properties of Milk Proteins

Due to the presence of many side chains and charged groups in the constitutive amino acids, proteins are amphiphilic molecules with a high reactivity. In this respect, milk proteins have unique properties that make them highly suitable for non-food applications, such as the production of biomaterials in the form of films and coatings. To further improve these properties, milk proteins can be intentionally modified by different approaches, with heat treatments and crosslinking being the most common.

### 3.1. Thermal Treatments

Thermal treatments are normally adopted by the dairy industry as part of the manufacturing processes that convert milk into safe food products. However, depending on the operating conditions, thermal treatments can induce changes to milk proteins in food mostly associated with the appearance of undesirable modifications in sensory and nutritional properties. These modifications, generally referred to as heat damage, have been the topic of extensive research for many years and further motivated the development of non-thermal processes that could avoid these drawbacks [28,29].

Differently, less attention has been paid to heat-induced changes that can be of interest for proteins destined for non-food applications. Heat can lead to protein unfolding, with the exposure of hydrophobic groups, aggregation, and flocculation. In general, the energy associated with thermal treatments is enough to destroy noncovalent interactions in the native structure, such as hydrogen bonds and hydrophobic interactions, thus modifying protein conformation and causing the exposure of hydrophobic amino acids residues and/or free sulfhydryl groups. β-Lg is most affected by thermal treatments among milk proteins. At a relatively low temperature (67–78 °C), this structural change is determined by unfolding, whereas at higher temperatures (>78 °C), the aggregation process becomes significant [30]. This process occurs in three steps: initiation, propagation, and termination. During the first step, the β-Lg dimers reversibly divide into monomers; subsequently, active monomers accumulate via an irreversible exposition of free sulfhydryl groups previously hidden inside the molecule. During propagation, aggregates form due to the formation of newly arranged disulfide bonds. Finally, in the termination step, active intermediates react to create larger aggregates without exposed/reactive SH groups [31].

Unlike β-Lg, α-La undergoes very limited aggregation due to the absence of free thiols in this protein. Both β-Lg and α-La in the unfolded status preferentially bind to the surface of casein micelles by reacting with κ-casein [26]. When severe heat treatments are applied, other inter and intramolecular interactions occur, leading to the formation of insoluble protein aggregates, also entrapping fat globules [32,33]. Aside from heating conditions, the protein concentration and pH of the medium also regulate the degree of whey protein binding to casein instead of self-aggregation, and this dramatically impacts milk protein functionality and behavior [34].

Although casein micelles themselves are very stable under heat treatment, exposure to high temperatures causes a change in the mineral equilibrium and, thus, can induce the partial disintegration of micelles [35]. Moreover, when milk proteins undergo thermal treatments in the presence of reducing sugars, such as lactose in milk, the α-amino groups of lysine residues can react with the reducing end of the sugar through the Maillard reaction [36]. Cardoso et al. [37] observed that susceptibility to the glycation of milk proteins individually mixed with different sugars in the dry state follows the order: α-La > β-Lg > β-casein. The extent of protein glycation influences many of their physico-chemical and functional properties, such as solubility, heat stability, gelation, and foaming properties. Structural changes arising from glycation generally determine a more flexible protein structure that enables molecules to move faster in an aqueous solution, improving their solubility as well as emulsion formation properties and foaming capacity. This last property is ensured by faster adsorption at the air/water interface. However, changes are very dependent on reaction conditions and pathways followed by the Maillard reaction. Consequently, controlled glycation could represent a useful tool for producing modified milk proteins with tailored properties [38].

### 3.2. Protein–Protein Crosslinking

Inducing covalent crosslinks at either intra- or intermolecular levels is another approach to modifying protein structure and functionality. Chemical, physical, and enzymatical treatments can be applied to induce protein crosslinking since proteins have several reactive groups (e.g., side chains of glutamine, lysine, tyrosine, cysteine) [39,40]. Disulfide bonds are naturally occurring crosslinks in whey proteins, which may rearrange when subject to thermal treatments, enzymatic catalysis, or oxidative conditions, with a significant impact on protein functionality [32]. The creation of inter- and intra-molecular disulfide bonds leads to the formation of a stronger, more rigid protein network with enhanced resistance to proteolysis [41]. The combination of extreme pH/high temperatures/low moisture conditions dramatically promotes the formation of protein crosslinks starting from a dehydroalanine residue intermediate, which derives from cysteinyl or phosphoseryl residues and further reacts with the ɛ-amino group of lysyl residues. Among these crosslinks, lysinoalanine is the most widely studied in milk proteins [34,42]. The competition existing between the Maillard reaction and dehydroalanine-mediated crosslinking towards lysine residues has been highlighted [34,43]. Oxidation phenomena are definitely less investigated in milk proteins. Nevertheless, the riboflavin-mediated photo-oxidation of milk proteins is rather well documented as a mechanism leading to the formation of crosslinks, such as di-tyrosine and di-tryptophan [44,45,46]. Huang et al. [47] studied the oxidative modification of a-La and reported that a moderate oxidation improved the emulsifying and foaming properties of the protein, while an extensive oxidation induced an increasing aggregation and impaired emulsion and foam stability. In the field of milk proteins, a particular importance is given to enzymatic crosslinking. This approach can be used to improve protein networking in order to reach specific features in terms of the stability, viscosity, and thickening of the protein matrix. Enzymatic crosslinking was studied for both casein and whey proteins [48,49,50]. In particular, the enzymatic crosslinking of casein with microbial transglutaminase (mTGase) has received much attention in recent years as a potential tool for improving the texture and properties of dairy products [48,51,52,53]. It has been demonstrated, for example, that sodium caseinate crosslinked by mTGase, when added to skim milk, increases the stiffness of the resulting acid gels [50]. This enzyme is able to catalyze the reaction between the γ-carboxy amide of glutamine and the ε-amino group of lysine residues, resulting in stable protein polymerization [54]. TGase crosslinking improved the water solubility, water vapor permeability, tensile strength (TS), wettability, and thermal stability of casein, whereas excessive crosslinking led to contrasting results [55].

## 4. Films and Coatings from Milk Proteins

**Figure 2:**
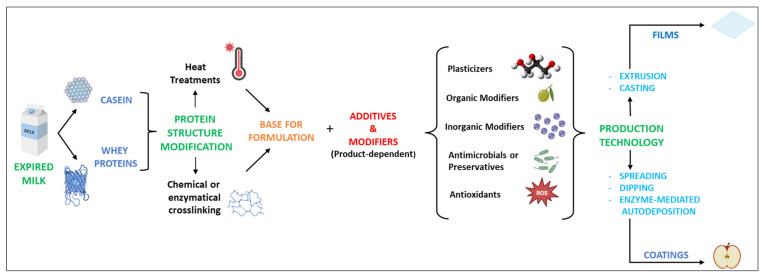
Overall approach to producing films and coatings from modified milk proteins.

Based on the aforementioned context, the remarkable characteristics of milk proteins can be consistently exploited in the production of materials as alternatives to plastic. The overall scheme, from the recovery of milk proteins to the production of films and coatings, is represented in Figure 2. In particular, the recovery of residual milk proteins from by-products or waste from the dairy industry chain can contribute to a more sustainable circular system. Food packaging materials are based on two different objects, films and coatings, which, apart from thickness, do not substantially differ in composition [56,57]. Nevertheless, they have different uses.

Films can be used to make pouches, wraps, capsules, or sleeves. Alternatively, coatings are directly applied to the surface of a material. Depending on its type, the coating might be removed from the coated material itself, but it is usually designed not to be discarded separately. Therefore, the coating is normally considered part of the final material. Edible films and coatings, in particular, are designed to be ingested together with the food. In this case, coatings are best applied on the surface of the food product by various approaches, as discussed later.

Casein itself possesses the ability to form films or coatings from aqueous solutions without further processing. This behavior is caused by its random-coil nature and the large number of electrostatic, hydrophobic, and intermolecular hydrogen bonds. Its additional useful properties include high thermal stability and biodegradability and the capability of forming micelles and binding a variety of ions and molecules. Acid-precipitated casein is easily turned into soluble caseinates through neutralization with an alkali (e.g., sodium, potassium, or calcium hydroxide). Nevertheless, single casein molecules in a water solution have a strong tendency to spontaneously aggregate depending on the solution concentration, the temperature, and the presence of Ca^2+^ ions [58]. Due to these features and behaviors, which can be easily tempered towards the required performances, casein in the form of caseinates is the most investigated among milk proteins as a material for producing films and coatings.

Whey proteins can be efficiently recovered from cheese whey, thus exploiting the potential of the most abundant waste of the dairy industry [59,60,61]. Globular proteins have the ability to unfold and form new polymeric structures via crosslinking under suitable conditions. These characteristics make whey proteins excellent raw materials for producing films as opposed to other film-forming biopolymers. Studies that focus on the development of films or coatings where caseinates or whey proteins represented a minor constituent are not considered in this review.

### 4.1. Films from Casein and Whey Proteins: Formulation and Production Methodologies

The scientific literature describes different formulations that can be used for milk protein film production, with different complexities depending on the adopted conditions and required properties. The type of protein used in building the film matrix is even more important. Although prepared under the same conditions, films obtained from either caseinate or whey proteins may have significantly different features, behaviors, and performances [62]. Sabil et al. [63] studied the characteristics of films simply obtained from water solutions of sodium caseinate and found 9.5% (*w*/*w*) to be the caseinate concentration that maximizes yield, TS, and thickness. A similar concentration was shown to be optimal by Picchio et al. [64]. However, due to the brittleness of the protein films, the addition of a plasticizer is usually recommended, as discussed below.

Essentially, protein films can be produced using two different methodologies: solvent casting or extrusion. Solvent casting is mostly used at a laboratory scale since it is easily implemented and does not require expensive equipment. Generally, the solvent casting process uses a pouring solution prepared by dispersing caseinate or whey protein isolates (5–10% *w*/*w*) in water or a blend of various solvents. A plasticizer is usually added, the pH value is adjusted to 8–9, and then the solution is heated up to 90–95 °C for 30 min for protein denaturation. The film is obtained by casting the solution on a smooth surface with an average casting volume of 0.1 mL/cm^2^ to obtain a thickness of about 100 µm. After casting, the film is normally dried at 20–30 °C and 40–50% RH in order to achieve the best structure before peeling off [60,65,66,67].

The extrusion process is normally used for large-scale industrial production due to the continuous operating conditions. Extrusion consists of a dry process that uses a co-rotating twin screw extruder. For caseinate film production, the powder is introduced into the first zone of the extruder using a gravimetric feeder. In the second zone, the plasticizer (usually glycerol) is introduced through a valve. Temperature progressively increases up to 70–75 °C from hopper to tie, which has approximately a 5 cm width and 1 mm thickness. The film thickness is about 500 μm, depending on the formulation. Residence time into the twin-screw extruder, with a flow powder rate of 2 kg/h and screw speed of 170 rpm, may vary between 2 and 6 min [68].

#### Casein and Whey Protein Pre-Treatments

As discussed before, protein crosslinking could be an interesting approach to improving film properties. However, it seems to be scarcely explored in the literature, specifically on the topic of milk proteins. Pereda et al. [69] evaluated the most relevant characteristics of casted films prepared from caseinate previously crosslinked with glutaraldehyde (GTA). The TS increased, compared with untreated caseinate, in samples prepared with more than 5% GTA. These results suggest the formation of a flexible network with high chain-segment mobility, promoting the diffusion of water molecules in agreement with the increased water vapor permeability (WVP). Nevertheless, improved behavior was also observed in films from caseinate crosslinked by thermal treatment [69]. The enzymatic crosslinking of caseinate was obtained using TGase, laccase or tyrosinase, and the derived casted films were characterized comparatively [70]. The tyrosinase-crosslinked film was proven to perform best in terms of solubility resistance, contact angle, and structural homogeneity. Picchio et al. [71] used tannic acid (TA) for crosslinking casein. As highlighted by FTIR analysis, the incorporation of TA produced an appreciable shifting in the amide I and amide II bands, with a relative increase in the former signal, which was attributed to a strong chemical bonding between the phenolic compound and the protein. Increasing the crosslinker concentration improved the TS but compromised the elongation at break (EAB). Adding 8% of TA produced a decrease in the moisture content of the film from 24% to 11%. Meanwhile, using 4% of TA led to a decrease in the WVP. A recent study [72] investigated the effects of treating sodium caseinate powder by dielectric barrier discharge (DBD) cold plasma at 7 kV and 10 kHz frequency on its structural, thermal and film-forming characteristics. The treatment resulted in an increased β-structure and reduced the random coil of the protein, with a positive impact on its interfacial activity in solution. The microstructure of the derived film, obtained using a casting method, was more uniform, and the mechanical strength improved compared to untreated caseinate film. Similar effects were observed by directly treating the casein film [67].

Commercial whey proteins that can be used as raw materials, mainly whey protein concentrates (WPC) and whey protein isolates (WPI) with a protein content of 60–80% or >95%, respectively, are normally derived from cheese whey and require a preliminary heat treatment for extensive denaturation. Notably, Abdalrazeq et al. [73] showed that films produced at pH 12 without heat treatment were more resistant, flexible and opaque compared to films produced at pH 7 with a heat treatment at 80 °C for 25 min. Similarly, casted films were obtained from a WPC previously submitted to different functionalizing pre-treatments: heat treatment, enzymatic crosslinking by TGase, and ultrasound [74]. Ultrasound-treated films showed increased TS and puncture strength, which could be caused by increased hydrophobic interactions of the unfolded protein molecules. The TGase-treated films had a lower water content, likely due to the reduced capacity of binding water for crosslinked proteins. Overall, heat-treated films had the best mechanical properties (TS, EAB, puncture strength). Díaz et al. [75] tested the effects of ultraviolet radiation (0.12–12 J cm^−2^ at 254 nm) applied to a film-forming WPC solution (8% protein) compared to those induced by heat treatment (80 °C for 20 min). UV radiation at the highest doses induced whey protein denaturation and aggregation comparable to heat treatment, with a-La being more extensively affected than β-Lg. The derived film had a higher TS, elastic modulus, puncture strength and lower solubility compared to control films. Aside from a rearrangement of disulfide bonds, these effects were also attributed to the concomitant oxidation of UV-absorbing amino acids, mainly tyrosine and tryptophan, which may form intermolecular covalent crosslinks.

### 4.2. Additives and Modifiers

In order to improve the properties of casein or whey protein films, compared to plastic films, some additives are normally required in the formulation to obtain the appropriate features, depending on the food packaging requirements. Furthermore, films can perform as an active packaging material by releasing selected substances beneficial to the packed food items [76,77]. Additives can be of natural origin (e.g., essential oils, plant extracts, phenols, enzymes) or inorganic (e.g., minerals, clays) and may have more than a single role or promote side effects. Depending on the added substance, the protein matrix may change in structure and thus in the properties of the derived film [68].

#### 4.2.1. Plasticizers

Plasticizers work by disrupting protein–protein interactions and, consequently, increasing the intermolecular free volume and the mobility of polymer chains, allowing good flexibility of the film. However, the sorption of water molecules into the film matrix increases the permeability coefficient and lowers the glass transition temperature [65]. Glycerol is the most widely used plasticizer for both casein and whey protein film production, especially for edible films [78]. Chevalier et al. [68] added different concentrations (13.2–24.2%) of glycerol in the formulation of a casein film and found a positive effect on its hydrophilicity (i.e., water contact angle values lower than 90°). In a whey-protein-based film, a glycerol concentration of 30% based on a protein gave the highest TS, while lower concentrations had no plasticizing effect [79]. Sorbitol is another studied plasticizer. The study of Brzoska et al. [65] highlighted that caseinate films containing sorbitol were stronger and less flexible compared to films containing glycerol. This was attributed to a couple of reasons: (i) polyol plasticizers interact with polar amino acid residues in biopolymers, resulting in a decreased intermolecular protein crosslinking; (ii) the high hygroscopicity of polyols facilitates the sorption of water molecules. Nevertheless, moisture barrier properties were much better in glycerol-plasticized films.

#### 4.2.2. Organic Modifiers

Plant extracts have a wide range of biological, antioxidant and antimicrobial activities and are generally recognized as safe (GRAS) products; thus, their use is also suitable for the fortification of edible films. The addition of essential oils primarily aims to modify the water barrier properties of the film by increasing the content of hydrophobic groups [80]. Pereda et al. [81] developed a sodium caseinate film supplemented with a linseed-oil-based resin (LOR). The WVP decreased, compared with the control, and was the lowest when the LOR addition was 10–15%. Alongside this effect, the addition of LOR caused a decrease in TS, whereas the EAB was not significantly affected. Alizadeh-Sani et al. [82] produced a casein film reinforced with rosemary essential oil and zinc oxide (ZnO) nanoparticles using glycerol as a plasticizer. Along with the expected antimicrobial effect of both additives, tested against some bacterial strains, the film demonstrated good water barrier and mechanical properties (flexibility and strength) and moisture resistance. Another type of active composite film, produced through solvent casting, was proposed by Ranjbaryan et al. [83] with an application in perishable food. The authors used sodium caseinate, two different amounts (2.5 and 5% *w*/*w* based on sodium caseinate) of cellulose nanofibers, and a cinnamon essential oil nanoemulsion. While the supplementation of cellulose nanofibers increased the crystallinity and reduced the porosity of the film, as confirmed by scanning electron microscopy (SEM), the further addition of essential oil induced a reduction in the WVP and moisture adsorption. The addition of cellulose in the form of nanocrystals (CNCs) has also been proposed to improve the mechanical properties of whey protein films [84]. The CNCs were incorporated into the water solution of whey proteins at a concentration of up to 8% (*w*/*w* based on whey protein) before casting and caused an increased TS and Young’s modulus of the film, while the WVP decreased. Including rapeseed oil in the formulation of a whey protein film decreased its water hydrophilicity, thus moisture content and solubility in water; on the other side, the film was more permeable to oxygen and carbon dioxide [85]. As expected, the WVP in the presence of rapeseed oil was significantly higher when the temperature was 5 °C instead of 25 °C [85].

Plant extracts are usually rich in phenolic compounds, many of which are attracting increased interest as natural free-radical scavengers (antioxidants). Brzoska et al. [65] investigated the effects of quercetin, an antioxidant compound, added to casein film formulations. Aside from other tested variables, a 100% radical scavenging activity was observed in undiluted extracts using the DDPH method. Furthermore, light transmittance measurements revealed that the incorporation of quercetin provided UV-light barrier effects at wavelengths between 300 and 400 nm. Interestingly, Fernandes et al. [61] proposed using a whey-protein-based edible film as a carrier of prebiotics, namely galacto-oligosaccharides (GOS) and xylo-oligosaccharides (XOS). In their study, these incorporated components reduced the melting point and WVP due to the overall increased hydrophobicity. Mechanical tests detected a lower TS and higher EAB. Based on previous evidence of the improved functionality of soy proteins resulting from the addition of γ-amino butyric acid (GABA), He et al. [86] tested the effects of introducing different concentrations of this amino acid in the recipe of a whey-protein-based film. The presence of GABA increased both the EAB and WVP of the film but reduced TS. The authors reported that GABA concentrations higher than 1% (*w*/*v*) impaired the hydration and thermal properties of the film.

#### 4.2.3. Inorganic Modifiers

This category includes a miscellaneous group of inorganic substances that have been added in casein or whey protein film formulation with the aim of improving selected properties. The advent of nanotechnologies has increased the use of inorganic nanoparticles in film preparation to enhance or modify their techno-functional characteristics. Nanoparticles of TiO_2_ [66] or ZnO [82] were incorporated in caseinate films at concentrations of 1–2% based on casein. Both TiO_2_ and ZnO nanoparticles increased the thermal stability of the film while decreasing its transparency and, acting against the plasticizer, also decreasing mechanical properties (strength, flexibility, stiffness). The WVP of the casein film significantly decreased due to the hydrophobic nature of the nanoparticles. In addition, the incorporation of water-impermeable particles into the casein matrix generated a tortuous pathway for vapor particles to move through [66]. Interestingly, caseinate films containing TiO_2_ and ZnO nanoparticles exhibited remarkable antimicrobial activity. The addition of halloysite in Na caseinate film production was investigated by Kajthunyakarn et al. [87]. Halloysite (HS) is a natural clay used as an additive and adsorbent in drug systems, acting as a drug carrier and allowing the sustained-release effect of drugs on dissolution. Films with different caseinate–HS ratios were prepared using a casting method and physico-chemical and mechanical properties, as well as drug permeability, were investigated. The results show that Na caseinate may interact with HS through hydrogen bonds between amine and amide groups of SC and hydroxyl groups of HS. The caseinate–HS film had a transparency similar to that of the unfortified film, while thermal stability was improved. The incorporation of HS caused a decrease in the puncture resistance and EAB of the films. Furthermore, the lowered water absorption resulted in delayed drug penetration through the film. A previous study by the same authors evaluated magnesium aluminum silicate (MAS), a layered montmorillonite clay, as an additive for modifying the properties of sodium caseinate films, achieving similar results [88].

Schmid et al. [89] investigated the effects of reactive additives, such as sodium sulfite (SS), sodium dodecyl sulfate (SDS) and urea in whey protein film and observed different complex interactions, often counteracting each other. The presence of SS did not affect the oxygen permeability and WVTR, even though the importance of hydrophobic interactions and hydrogen bonds increased. Urea caused an increase in WVTR and oxygen permeability but without intermolecular changes. SDS was the most effective additive for permeability reduction at concentrations as low as 1%.

#### 4.2.4. Antimicrobial Agents and Preservatives

The antimicrobial activity of agents incorporated into foods may be reduced by compounds within the food matrix. Contrastingly, the incorporation of antimicrobial compounds into the packaging in contact with food may result in a selective and steady migration of these compounds from the packaging material to the food surface, where they subsequently disperse [57].

Alongside the array of aspects related to the actual effectiveness of the antimicrobial agent included in the material, other factors must be considered regarding its possible interference with the physical or mechanical properties of the film. The effects of plant extracts and essential oils on active casein or whey-protein film formulation have already been discussed. Colak et al. [90] successfully produced a caseinate film containing hen egg lysozyme (E1105), a food additive that exhibits antibacterial activity against most Gram-positive and some Gram-negative bacteria. Under the optimized conditions for the extrusion process (twin-screw extrusion at 65 °C and a glycerol content of 25 or 20%), the relevant properties of the film were not affected by the presence of lysozyme, and the enzyme retained about 26% activity. Lysozyme was also incorporated in a whey protein film matrix, either as a free molecule or complexed with polyacrylic acid [91]. The complexed form proved to be released more slowly than the free form (up to 500 h instead of 24), creating a long-term antimicrobial effect against *Listeria innocua*. Chevalier et al. [92] investigated the antimicrobial activity against *Escherichia coli* of a film obtained from rennet casein mixed with a potassium sorbate and different natural waxes (beeswax, Candelilla, and Carnauba wax). A twin-screw extrusion process was adopted. The sorbate proved to inhibit *E. coli* even at low concentration (2%) and over 20 days of storage at 15 °C, confirming that the casein matrix is a good preservative carrier. Furthermore, the addition of 10% of potassium sorbate revealed a plasticizing action on the film, but only beeswax decreased the WVP by 20%. Yoplac et al. [93] developed a sodium caseinate film, plasticized by sorbitol (Sb), with the incorporation of citral microparticles (CMs) as an antimicrobial agent. The authors used a response surface methodology to optimize the proportion of components in the formulation and other parameters, such as opacity and elastic modulus. Using this methodology, based on FT-IR and MID (mid-infrared) spectroscopy measurements, the optimal conditions were NaCas:Sb = 1:0.91 and NaCas:CMs = 1:0.95. However, SEM revealed discontinuities and micro-holes on the surface of the film, likely due to the incorporation of citral microparticles and the insufficient elimination of air bubbles during mixing, highlighting the crucial role of film settling conditions aside from the optimization of the formulation. Differently, Agudelo-Cuartas et al. [94] obtained a homogeneous structure in a whey-protein-based-film where both natamycin and an oil-in-water nanoemulsion of α-tocopherol were added. Aside from an effective inhibitory activity against various microbial species, inclusion induced an increase in the EAB, opacity, UV–vis light barrier, and WVP of the film. Vanden Braber et al. [95] developed a whey protein film added with low quantities of a water-soluble derivative of chitosan (WSCh) and neutralized with sodium tripolyphosphate, which showed excellent antifungal activity against *Aspergillus niger* (100% inhibition).

The incorporation of viable lactic acid bacteria strains into the film for the in situ production of bacteriocins is a novel approach. In the study by Mozaffarzogh et al. [96] probiotic strains of various *Lactobacillus* spp. and *Bifidobacterium* spp., all showing an inhibitory effect towards biogenic amine formation, were individually incorporated in sodium caseinate films containing carboxymethyl cellulose (CMC) and intended to extend the shelf life of fresh trout fillets. Two separated solutions of NaCas–glycerol and CMC–glycerol were mixed in 1:1 ratio; then, each strain was incorporated at a final concentration of 9 log CFU/mL, and the mixture was cast in a Petri dish. Although still preliminary, the results were encouraging since the film provoked a 2-week delay in microbial growth in fish fillets, compared with the control, due to the migration of the probiotic strains from the film to the fish [96]. The bacteriocin-producer *Lactobacillus curvatus* 54M16 was incorporated in a whey-protein-based film and tested against *Listeria innocua* C6 [97]. The presence of bacterial cells improved the elasticity and the percentage of EAB of the film while having no effect on WVP. Sogut et al. [98] observed that probiotic lactic acid bacteria were more stable in films obtained from blends of whey proteins and carrageenan than in films obtained from whey proteins only.

An overview of the above-described studies is reported in Table 2. Although most of these studies focused on innovative approaches, very few included testing possible applications in food packaging. To the best of the authors’ knowledge, no studies thus far have explored an industrial application of milk protein films in the food packaging sector.

**Table 2:** Overview of the recent studies aimed at developing milk-protein-based films.

Type of Material	Aims	Formulation	Production Conditions	Main Results	Reference
Edible Casein Sheets	Develop films from different casein types. Evaluation of glycerol (Gly) concentration on the extrusion process	Rennet casein/acid casein/sodium caseinate (NaCas); Gly 13.2%/24.2% *w*/*w* on protein.	Extrusion + conditioning at 23 °C/50% relative humidity (RH) for 48 h	Gly concentration, RH, and type of casein are relevant parameters. The higher the Gly concentration, the higher the susceptibility to water on the film.	[68]
Edible Casein Film	Study of properties of films obtained using different casein concentrations	Casein 7.5/8.5/9.5% *w*/*w*; Gly (amount not indicated).	Casting + drying (oven) at 50 °C for 5 h + conditioning at 27 °C for 2 days	Higher casein concentration (9.5%) showed higher yield, increased thickness, and tensile strength (TS).	[63]
Edible Casein Film	Use of dielectric barrier discharge (DBD) cold plasma technology for improving casein film properties	Casein; Gly; 0.5 M NaOH; Rate of 5:1:30 by weight.	Casting on polyacrylic plates + drying at 35 °C/50% RH for 48 h Films loaded onto quartz reaction device, treated with DBD-50 Plasma Reactor at different conditions of voltage and time	DBD cold plasma improved mechanical and barrier performances: increased TS, elongation at break (EAB); decreased water vapor permeability (WVP).	[67]
Casein Film	Develop a NaCas-based film added with linseed oil resin (LOR)	NaCas 2.5% *w*/*v*; LOR 0.05/0.1/0.15/0.2% *w*/*w* on protein.	Casting on Teflon Petri dish + drying at 35 °C for 10 h + conditioning at 23 °C-50% RH for 3 days	LOR decreased film WVP; minimum was reached with 10–15% addition. Decrease in tensile modulus (TM) and TS; EAB remained stable.	[81]
Casein Film	Casein crosslinking with tannic acid (TA) to obtain a film with better physico-chemical properties for food packaging	Casein 10% *w*/*w*; Carbonate buffer; Gly 50% *w*/*w* on protein; TA 4/8/10/15/20% *w*/*w* on protein.	Casting on silicon molds + drying at room temperature (RT) for 7 days	TA was a good crosslinker for casein, as confirmed by FTIR. Protein network was modified by concentration of phenolic acid. Increased TS and decreased EAB; TA improved water resistance of the film.	[71]
Active Casein Film	Develop a NaCas-based film including microencapsulated citral as antimicrobial	NaCas 5% *w*/*w*; Sorbitol (Sor) NaCas:Sor = 1:0.5 to 1:1.5 *w*/*w*; Citral microparticles (CM) NaCas:CM = 1:0.5 to 1:1.5 *w*/*w*.	Casting + drying at 25 °C-55% RH for 24 h	Best film resistance at NaCas:Sor = 1:0.91 and NaCas:CM = 1:0.95. Mechanical properties were acceptable, but SEM revealed surface discontinuity.	[93]
Active Casein-CMC film	Develop a carboxymethyl cellulose (CMC)-NaCas-based film containing 5 probiotic bacterial strains and application on trout fillets.	NaCas/Gly (5 g + 3.75 mL in 100 mL water); CMC/Gly (1 g + 0.75 mL in 100 mL water); Solutions mixed 1:1; Added species: *L. acidophilus*/*L. reuteri*/*L. casei*/*L. rhamnosus*/*B. bifidum.*	Casting on Petri dish + drying at ambient conditions for 48 h	During 14-day refrigerated storage, delay in both spoilage bacteria growth and formation of biogenic amines compared to the control. Films containing *L. acidophilus* were most effective.	[96]
Active Casein Film	Develop casein active films reinforced with ZnO nanoparticles and rosemary essential oil (REO) and properties investigation	NaCas 8% *w*/*w*; Gly 4.5% *w*/*w*; ZnO NPs; REO 1–2% *v/w* on protein.	Casting on Petri dish + drying in oven at 30 °C-50% RH for 24 h + conditioning at 25 °C-50% RH	ZnO + REO increased barrier, mechanical properties, and humidity resistance. WVP reduction, strength and flexibility increase. Good antimicrobial activity against tested bacteria.	[82]
Active Casein Film	Develop a NaCas film added with cinnamon essential oil nanoemulsion (CEO-NE) as antimicrobial and cellulose nanofibers (CNF) as mechanical reinforcement	NaCas 4% *w*/*v*; CEO-NE; CNF; Gly 37.5% *w*/*w* on protein.	Casting on polystyrene Petri dish + drying at 25 °C-50% RH for 48 h	Presence of CNF increased film crystallinity, SEM revealed decreased surface roughness. Decrease in both WVP and adsorption of humidity. Improved mechanical properties and controlled release CEO-NE due to CNF; CEO-NE showed a little antimicrobial activity.	[83]
Active Casein Film	Study of effects derived from type of plasticizer and lipid concentration included in NaCas-based films. Investigation of quercetin (QC) antioxidant effect	NaCas 10% *w*/*w*; Gly/Sor 66% *w*/*w* protein; Oleic acid 35% *w*/*w* protein; Beeswax (BW) 0/6.6/10/15/20% on DM; QC 1% *w*/*w* total dispersion.	Casting on Petri dish + drying at 23 °C-40% RH for 3 days	Water vapor transmission rate (WVTR) was significantly lower in Sor-plasticized films. Adding 10% BW strongly decreased WVTR in the presence of Gly. Sor improved TS and Young’s modulus (YM) but worsened EAB. QC extracts showed an effective radical scavenging activity.	[65]
Edible WP film	Investigating and testing properties of WP edible films	WPI at 7/8/9/10% *w*/*w*; Gly at 30/40/50/60% *w*/*w* on protein.	Casting on petri dishes + drying at 25°-50% RH × 24 h + conditioning at 25°-53% RH × 48 h before testing	Increasing protein concentration leads to lower opacity and moisture adsorption of films and increased TS and EAB. Films with higher glycerol concentration showed weakened mechanical resistance and higher moisture adsorption rates.	[79]
WP Edible Film	Improve the physical characteristics of whey protein isolate (WPI)-based film using γ-aminobutyric acid (GABA)	WPI 6% *w*/*v*; GABA 0.0/0.1/0.5/1.0% *w*/*v*; Gly 1:25 *w*/*w* on protein; pH = 8.	Pouring on perspex sheet + drying overnight	Films made with GABA had an increased EAB and WVP and decreased TS and light transmittance. Lowest concentrations of GABA enhanced hydration and thermal properties.	[86]
Active WP Edible Film	Develop antifungal films from WPI added with low quantities of a water-soluble derivative of chitosan (WSCh)	WPI 5% *w*/*v*; Na tripolyphosphate 2 mM; Gly 1.8% *w*/*v*; WSCh 1.5/3% *w*/*w* on protein.	Casting on polystyrene Petri dish + drying at 60 °C for 3 h in oven + conditioning at 20 °C-58% RH for 48 h	WSCh acts as a crosslinking agent through H bonds, causing a decrease in EAB and solubility. WPI/WSCh films had excellent fungistatic activity and barrier effects. *Aspergillus niger* was 100% inhibited, while *Penicillium roqueforti* was more resistant.	[95]
WP Edible film	Develop an edible film using WPC previously treated with heat, high-power ultrasound (US) and/or crosslinked with microbial TGase	WPC 8% *w*/*w* + Gly 50% on protein + different treatments: - no treatment - crosslinking - heat treated (HT) - HT+ crosslinking - US for 15 or 60 min - US + crosslinking	Casting on Plexiglass Petri dish + drying in oven at 35 °C for 18 h + conditioning at 50% RH for 96 h before peeling + 20 °C-50% RH for 48 h	US treatment slightly decreased WVP and increased puncture strength and TS of the films. TGase crosslinking increased puncture deformation values and affected film color. Heat-treated films had the best mechanical properties and thus were tested as separation material for cheese slices.	[74]
Active WP film	Develop a film based on WPC added with natamycin (Nat) and/or α-tocopherol (αTOC) nanoemulsion	WPC 8% *w*/*w* + Gly + - control - αTOC 2% - Nat 300 ppm - αTOC 2% + Nat 300 ppm	Solvent casting on polystyrene Petri dishes + drying in oven at 30 °C for 12 h + conditioning at 25 °C-58% RH for 7 days	Addition of αTOC and Nat significantly decreased TS and EM and increased EAB. Opacity, UV light barrier, and WVP of composite film also increased. Antioxidant activity and antimicrobial effect against *C. albicans*, *P. chrysogenum*, and *S. cerevisiae* were evidenced.	[94]
Active WP film	Develop a WPI film carrying a controlled release system for lysozyme (LYZ) based on pH-responsive polyacrylic acid (PAA)/LYZ complex incorporated within the matrix	WPI 5% *w*/*w* + Gly 50% + - free LYZ - PAA/LYZ complex - free LYZ:PAA/LYZ complex = 1:1 - Total LYZ amount was constant	Casting on polypropylene substrate + drying overnight at 25 °C-40% RH	PAA molecular weight affected the surface charge and hydrophilicity of the films. Incorporating PAA/LYZ complex into film extended its release time up to 500 h due to a low diffusivity. A 5.7 log reduction in bacterial population within 72 h was observed. Free LYZ was hardly effective against *Listeria innocua*.	[91]
WP film reinforced with CNCs	Develop a WPI film reinforced through addition of cellulose nanocrystals (CNC) extracted from sugar bagasse	WPI 5% *w*/*w*; Gly 50% dry matter; CNC from 0% to 8% *w*/*w*.	Casting on Teflon mold + drying in incubator at 50 °C for 15 h + conditioning 25 °C- 50% RH for 48 h	Lightness and transparency of the films decreased with increasing WPI content. CNC increased film hydrophilicity (lower water contact angle values), increased TS and YM, and reduced the WVP. Oxygen permeability did not change.	[84]
WP film added with Sodium Solfite, SDS and Urea	Evaluate effects of reactive additives sodium sulfite (SS), sodium dodecyl sulfate (SDS), and urea on properties of WP films	WPI 10% *w*/*w*; Gly 66.7% *w*/*w* on protein; Additives (SS, SDS and urea) 1 to 20%.	Casting on Petri dish + conditioning at 23 °C-50% RH for 9 days at least	SS led to increased number of hydrophobic interactions and H bonds and slightly decreased number of disulfide bonds. O_2_ permeability decreased from 68 to 46 cm^3^ with 1% SDS addition. WVTR decreased with 20% SDS addition.	[89]
WP edible films modified with UV radiation	Evaluate effects of UV radiation on WPC treating either the film-forming solution or the film	WPC 8% *w*/*w*; Gly 50% *w*/*w* on protein; UV radiation doses: 0.12 J cm^−2^, 4.0 J cm^−2^, 12.0 J cm^−2.^	UV exposure in a stainless-steel exposure chamber of a microprocessor-controlled UV radiation system	UV radiation of solutions increased free-SH groups and induced formation of aggregates. Derived films showed significantly higher TS, puncture strength and puncture deformation. Solubility was lower than for finished films exposed to UV. When UV was applied to solutions, films were more tinted.	[75]
WP edible film added with rapeseed oil	Develop WPI films added with rapeseed oil (RSO)	WPI 8% *w*/*w*; Gly 50% *w*/*w* on protein; RSO 0/1.0/ 2.0/3.0% (*w*/*w*).	Casting on Petri dish + drying at 25 °C-50% RH for 24 h + conditioning at 25 °C-50% RH for 48 h before testing	Presence of RSO decreased film WVP and water hydrophilicity, increased permeability to O_2_ and CO_2_. WVP and diffusion coefficient values were higher for films stored at 5° C than at 25° C.	[85]
WP film produced under alkaline conditions	Develop WP-derived materials obtained under alkaline conditions and with no heat-treatment	WPI 1% *w*/*v*; Gly 30/40/50% *w*/*w* on protein; Poliglicolic acid; NaOH 0.1 N to pH 7/12.	Casting on Petri dish + drying at 25 °C-45% RH for 48 h	Casting films containing either 40 or 50% of Gly at pH 12 led to the production of more resistant and flexible materials than at pH 7. Opacity was also higher for films obtained at a pH of 12.	[73]
Active WP film	Develop a WPC active coating, incorporated with seaweed (*Fucus vesiculosus)* extract (SWE) as antioxidant	WPC 8% *w*/*w*; Gly 8% *w*/*w*; SWE 1% *w*/*w*.	Casting on aluminum foil surface + drying at room temperature (RT) for 3–4 days. Chicken breasts were vacuum packaged with the films.	Presence of SWE improved thickness, TS, and EM of the film. The active film also inhibited lipid oxidation at the surface of chicken breast for up to 25 days of storage.	[99]
Active edible WP film	Study viability and antimicrobial activity of bacteriocin-producing lactic acid bacteria (LAB) incorporated in a WP film in presence or absence of nutrients	WP 13% *w*/*v* + Inulin 4% + Gelatin 6% + Gly 6% + - Control, no addition - *L. curvatus* 4% *v/v* in m-MRS broth - *L. curvatus* 4% in distilled water	Casting into Petri dishes + drying at 30 °C-50% RH for 24 h	The presence of LAB reduced viscosity of film-forming solutions and improved elasticity and EAB of film. WP-based films ensured high viability of LAB strains during 28-day storage at 4 °C. Adding MRS broth slightly affected the viability but was needed to achieve a good antimicrobial activity against *L. innocua*.	[97]
WP film added with XOS and GOS	Develop a WP film added with xylooligosaccharide (XOS) and galactooligosaccharide (GOS).	WP: amount depends on prebiotics concentration; Gly 30% *w*/*w*; XOS, GOS 0–10-20–30% *w*/*w*; Total film solid conc.: 10%.	Casting onto Petri plates + drying at 27 °C for 24 h + conditioning (vacuum) at 75% RH for 48 h before testing	XOS and GOS addition resulted in films with similar structure (cross-sectional SEM), with lower TS and higher EAB than control films. Prebiotics reduced WVP, despite the higher hydrophobicity evidenced by contact angle reduction.	[61]
WP film added with probiotics	Develop a composite film made with WP and carrageenan (CA) as a carrier of probiotic strains	WPI at 5% *w*/*w*; Gly 50% *w*/*w* on protein; Addition of strains; Same trial with: CA 1% *w*/*w*; Gly 35% *w*/*w* on CA powder and with blended solutions.	Casting onto Teflon-coated plates + drying at RT + conditioning at 25 or 4 °C-53% RH, before testing	Significant decrease in cell counts observed for all strains in both WPI and CA films during storage at 25 °C, whereas counts were stable in blended films. Multi-strain cultures presented the same behavior. Incorporation of probiotic bacteria influenced WVP and color values of films, decreasing TS and EAB.	[98]

### 4.3. Coatings from Casein and Whey Protein: Formulation and Production Methodologies

The production of coatings, starting from biodegradable raw materials such as proteins, is a still-developing sector, and the vast majority of coatings are produced using a synthetic material coupled with a biopolymer. As stated above, this review does not consider mixed materials.

Both caseinates and whey proteins can be used to produce coatings, with the latter being more frequently preferred in the case of edible coatings due to its absence of taste, superior nutritional value and lower price [60]. Both protein types shall be used in combination with other compounds that improve mechanical behavior and minimize sensitivity to the moisture of the protein matrix [100,101]. Protein-based coatings have the disadvantages of brittleness and poor water resistance. In this regard, Picchio et al. [102] proposed to modify casein by conjugating methacrylic groups to side amino groups in the protein chain, thus increasing its hydrophobicity. The chemically modified casein polymerized more efficiently, and the obtained material had enhanced technological properties, in particular, water resistance.

Compared to film preparation via a casting technique, the coating technology offers important advantages. Coatings are normally layered on a plastic or cellulosic support, and thus their formulation can be simpler. Recently, edible coatings were developed that can be directly applied to the surface of perishable foods. Depending on their formulation, the coating creates a barrier able to prevent moisture loss, mechanical damages, oxidation, or microbial spoilage [103]. This implies, however, that direct contact between the coating and food surface without air gaps is obtained. The film-forming dispersions for direct coatings are also widely used in the pharmaceutical industry to protect capsules and tablets.

#### 4.3.1. Coating Application Methods

In coating applications, different approaches can be used, depending on the functionality. Spraying or spreading the formulated solution uniformly on a surface with an applicator is a relatively common method. A suitable method for edible coatings is to dip a food product into a solution for a short time, usually a few minutes. After dipping, a dripping step is required for the removal of excess liquid, and then a final drying step allows the coating to cake. This final step is usually carried out at room temperature so that the coating performance is not affected. Furthermore, dipping technology is particularly suitable for food products that may be affected by high temperatures. Enzyme-mediated autodeposition has been proposed as an alternative to these conventional techniques for casein coating production. This approach exploits the ability of casein micelles to lose solubility after enzymatic cleavage via chymosin [104,105]. The enzyme (chymosin) is immobilized on a support, which is further coated with destabilized casein particles. Due to the enzyme immobilization via spacer molecules, it is possible to carefully control the process. The results of this study have revealed that the use of immobilized single-chymosin molecules can form a continuous casein monolayer.

#### 4.3.2. Additives and Modifiers

Additives added in the formulation of coatings vary depending on the type of product (inert supports or food items) that is coated. Dávalos-Saucedo et al. [106] developed a whey-protein-based coating intended for application on eggshells, thus increasing the shelf life of eggs. Essentially, pectin (20%) was crosslinked to the protein via TGase in the film-forming solution. The obtained coating reduced both the weight loss of eggs and post-wash bacterial penetration. The same approach was successfully used for coating roasted peanuts to prevent oxidation [107]. Decreases in peroxide value and water content were observed in the coated product compared with the uncoated one. Valentino et al. [101] developed coatings with good antioxidant properties by adding rosemary oil or gallic acid to a sodium caseinate solution (4% *w*/*w*). Minimally processed fennels were coated via the dipping process, and the dry layer was 0.7–6 µm thick. Other additives, such as tea polyphenols, lemongrass essential oil (LGEO) and lemon essential oil (LEO), were used as antioxidants in whey protein coatings by Ming et al. [108] and Galus et al. [109], respectively. Tea polyphenols increased both the zeta-potential and surface hydrophobicity of the solution. Pieces of fresh-cut apple coated with this solution demonstrated a reduction in browning, compared with the uncoated control, with the increased addition of polyphenols ranging from 0.1% to 0.5% (*w*/*v*). The same antioxidant activity was achieved by adding LEO and LGEO in a whey protein coating applied to fresh-cut pears. In the study performed by Mileriene et al. [110], a liquid whey protein concentrate (12.34% dry matter), was prepared from fresh sweet whey by ultrafiltration and the addition of sunflower oil (4% *w*/*w*) and cinnamon extract (6% *w*/*w*), and with glycerol as the plasticizer. It was then homogenized and immediately applied on the surface of fresh acid-curd cheeses (350 g each) via dipping. The results highlight a strong antimicrobial effect of the coating during 1 month of cheese storage, with decreasing counts of yeasts and molds and no significant changes in composition and sensory properties.

Natamycin (E235) is commonly used for the surface treatment of cheeses and raw sausages against the growth of yeasts and molds. This compound was added (0.07% *w*/*w*) to an acid casein (7.5% *w*/*w*) solution that was tested for coating Kashar cheese [111]. The antimicrobial activity was effective throughout a 90-day ripening period. Similarly, natamycin was added in a whey-protein-based coating, also containing lysozyme-xantan gum conjugate, which was layered on an ultrafiltrated white cheese [100]. This coating avoided the growth of *Penicillium chrysogenum* but also reduced the growth of pathogenic bacteria such as *Escherichia coli O157: H7* and *Staphylococcus aureus*, which were intentionally inoculated on the cheese surface. Furthermore, the authors observed a reduced moisture loss of coated cheese compared to the uncoated control during a 60-day ripening, with no effect on taste.

The antimicrobial activity of ZnO was exploited with the assembly of casein-based composite coatings, including hollow ZnO nanospheres [112] or ZnO nanoparticles [113,114]. Inclusion usually requires both chemical and mechanical action (prolonged stirring or ultrasonication) and the addition of a casein emulsifier/stabilizing agent, such as caprolactam. In general, the inclusion of inorganic nanoparticles, such as ZnO, TiO_2_, and SiO_2_, in casein-based coating materials also improves hydrophobicity, mechanical properties, covering ability, and self-cleaning properties under the action of UV light [113,114].

As previously described for casein or whey-protein-based films, the use of probiotics in a formulation of coatings that are capable of producing antimicrobial agents in situ seems to be a promising approach. Pereira et al. [115] separately incorporated two different commercial probiotic strains (from *Bifidobacterium animalis* or *Lactobacillus casei*, respectively) in the whey protein solution, which was then used for coating ham slices. Both coated and uncoated (control) ham slices were modified and atmosphere-packed. The main outcome of the storage trials was a decrease in water loss in coated ham compared to the control, with no color changes. The inhibition of common spoilage bacteria was achieved using viable probiotic cell numbers remaining at ca. 10^8^ CFU/g during 45 days of storage at 4 °C. Such high levels of the tested probiotic species can also be directly beneficial for human gut flora. Wang et al. [116] developed a whey protein coating (0.5% *w*/*w*) where carvacrol was added to preserve fresh-cut cheddar cheese in synergy with whey protein nanofibrils (WPNFs). WPNFs, prepared via a 10 h filtrate incubation at 80 °C under prolonged magnetic stirring, improved technological and functional properties, such as foaming and emulsifying properties, self-supporting gelling ability, and viscosity. The obtained coating had a smoother and continuous film surface with a higher antimicrobial and antioxidant activity with respect to traditional WP coating.

In summary, different from what was observed for the films, several recent studies proposed applications of milk-protein-based coatings in food packaging. An overview of the aforementioned studies, focusing on the latest advances in this field, is shown in Table 3.

**Table 3:** Overview of the recent applications of milk-protein-based coatings in food packaging.

Type of Material	Aims	Formulation	Production Technology	Application	Main Results	Reference
Antioxidant WP coating	Develop a WP coating incorporated with tea polyphenols (TP) for preserving fresh-cut apples	WP 5–10% *w*/*v*; Glycerol (Gly) 50% *w*/*w*; TP powder 0.1–0.5% *w*/*v*.	Dipping for 5 min + draining for 10 min + storing at 20 °C for 24 h	Fresh-cut apples	Antioxidant activity increased with increasing TP concentration. Coated slices showed lower browning during 24 h storage.	[108]
WP edible antioxidant coating	Develop WP edible coatings with incorporated lemon (LEO) and lemongrass (LGEO) essential oils for preserving fresh-cut pears	WPI acq. Solution 8% water + WPI; Gly 50% *w*/*w* dry basis; LEO 1% *w*/*w*; LGEO 0.5% *w*/*w*.	Dipping for 2 min + draining on a filter paper + packing under modified atmosphere + storing at 4 °C-80 % relative humidity (RH) for 28 days	Fresh-cut pears	Presence of LEO and LGEO reduced O_2_ and CO_2_ permeability of the film. Coating caused reduction in color changes and loss in hardness of pear slices.	[109]
Active casein coating	Develop an active casein coating by adding antioxidant substances. Investigate effects of NaCas concentration on coating properties	NaCas 4/8/10/12/14% *w*/*v*; Gly 0.4/0.8/1.0/1.2/1.4% *w*/*v*; Gallic Acid 0.005% *w*/*v*; Rosemary Essential Oil 1.5% *v/v*.	Dipping for 2 min + draining on metallic grids for 10 min	Fresh fennels	Thickness of dry coating was 0.6–7.2 µm, depending on NaCas concentration, with good antioxidant properties. Water vapor permeability (WVP) was highest for films with 4 and 14% NaCas.	[101]
Composite WP–pectin coatings	Develop WP–pectin complex coating enzymatically reticulated by TGase and applied to eggshells to increase egg shelf life and to roasted peanuts to prevent oxidation	WPI 4.8% *w*/*v*; Sor 2.4% *w*/*v*; Pectin 4.8% *w*/*v*; Final solution: WPI/PEC-4:1; TGase 8 U/g of WPI.	Eggs: Dipping for 1 min in the solution + drying for 10 min at room temperature (RT) Peanuts: Dipping for 10 s + drying at RT for 10 min + packing in sealed low-density polyethylene bags	Eggshells, Roasted peanuts	Eggs: Coating maintained a higher yolk index and albumen CO_2_ content, reduced weight loss and increased albumen and yolk pH. Compared to uncoated eggs, eggshell strength was higher and post-wash bacterial penetration was lower. Peanuts: Coating reduced the peroxide value. TGase reticulation decreased WVP and thus seed water absorption.	[106,107]
Antimicrobial WP coating	Develop an edible active coating for improving shelf life of fresh-curd cheese	LWPC (liquid WP conc.) 5.0% *w*/*w*; Gly 5% *w*/*w* on protein basis (pb); Guar Gum 0.7% *w*/*w* pb; Tween 0.2% *w*/*w* pb; Sunflower oil 4% *w*/*w* pb; Cinnamon CO_2_ extract 6% *w*/*w* pb.	Dipping into coating solution for 3 s + drying on perforated metal trays at 12 °C for 30 min + vacuum packing	Fresh-curd cheese	After 31-day ripening, coating had no effects on cheese moisture, color, texture, flavor. Coating had strong antimicrobial effect, and thus efficiently extended cheese shelf life compared to uncoated control cheese.	[110]
Antimicrobial casein coating	Investigate the efficacy of a casein/natamycin (NTM) coating for the control of mold growth on cheese surface	Acid casein 7.5% *w*/*w*; Gly 2.5% *w*/*w*; NTM 0.07% *w*/*w*.	Dipping cheese into the solution twice for 60 s + leaving to drip for 2 h + draining at 22 °C for 1 h	Kashar cheese	The casein/NTM coating suppressed mold growth during 90-day ripening without adverse effects on cheese quality but a slight decrease in ripening.	[111]
WP nanofibril-based antimicrobial and antioxidant coating	Develop a coating using WPI nanofibrils (WPNFs), incorporating carvacrol (CA) for preserving fresh-cut cheddar cheese	WPNFs 5% *w*/*w*; Gly 5% *w*/*w*; CA 0.5% *w*/*w*.	Dipping cheese pieces into the solution for 60 s + draining at RT for 30 min + storage at 4 °C for 10 days	Fresh-cut cheddar cheese	WPNFs-CA coating had smooth and continuous surface, promoting lower weight losses and better textural properties in cheese. Antimicrobial activity was higher than in traditional films due to CA.	[116]
Active antimicrobial WP edible coating	Develop WP coating incorporated with probiotics and investigate its antimicrobial activity on sliced ham preservation	WPI 10% *w*/*v*l Gly 5% *w*/*w*l Strain centrifuged pellet 5% *w*/*w* (*B. animalis* Bb-12^®^ or L. casei-01).	Slice immersion for 2 min in the solution + draining for 30 sec + storage at 4°C for 45 days	Sliced ham	Coating decreased water loss of ham without changes in color. Probiotics incorporated in coating inhibited growth of *Staphylococcus* spp., *Pseudomonas* spp., *Enterobacteriaceae* and yeasts/molds during storage.	[115]
Active edible WP film/coating	Develop a coating containing a nanoemulsion of cocoa–liquor (nCL) for improving physical and functional properties and prolonging the shelf life of muffins	WPC 8% *w*/*w*l Gly 5/6/7% *w*/*w*l nCL 0/1/2% *w*/*w* (different microfluidization conditions).	Dipping muffins into the solution for 5 s + drying at 40 °C for 15 min	Muffins	nCL modified the secondary structure of the WP (FTIR), decreasing mechanical properties, solubility and WVP of the film. Moisture loss during storage at 20–50% RH was lower for coated muffins compared to the uncoated control.	[117]

### 4.4. Relevant Properties of Milk-Protein-Based Films and Coatings and Future Developments

As widely discussed previously, films and coatings from milk proteins can protect food from changes in moisture, the loss of volatiles, oxidation, and microbial spoilage. For food packaging applications, the gas and vapor barriers are certainly the most sought-after properties for the preservation of sensitive foods: common food packaging polymers with high O_2_ barrier properties have gas transmission rates in the range of 0.1–0.5 cm^3^ m^−2^ d^−1^ bar^−1^ at 23 °C and 0%RH and vapor permeability (WVP) in the range of 1–5 × 10^10^ g H_2_O Pa^−1^ s^−1^ m^−1^ at 23 °C. Many biopolymers have interesting oxygen barriers but are more sensitive to water vapor. It is, therefore, necessary to combine the properties of different materials using multilayer and coating structures [12]. In this latest research, the deposition of very thin films and coatings of biopolymers on the surface of plastics and paper can reduce permeability to gas and moisture [118,119], delay the migration of contaminants [120,121] and improve the surface properties of the materials on which they are deposited [122].

Whey protein layers with an optimized plasticizer content present good oxygen and moisture barrier properties [123]. The crosslinked protein network provides an oxygen barrier in the same range of ethylene vinyl alcohol (EVOH) with average ethylene contents (<2 cm^3^ m^−2^ d^−1^ bar^−1^ at 23 °C and 0%RH), thus confirming its potential application in multilayer structures. In this way, biopolymers can contribute to the replacement of more expensive oil-based barrier resins, such as polyvinylidene chloride (PVDC), ethylene vinyl alcohol (EVOH) and aromatic polyamide (MXD6), commonly used in multilayer films. The same authors highlighted that the deposition of whey proteins on a PET film guarantees high transparency (i.e., light transmission >95%), temperature stability and interesting mechanical performance, as well as good adhesion to the substrate [123]. In a more recent study, a whey protein solution was layered on a PET film that was previously treated to improve the interfacial compatibility of the two layers. The final material, also including layers of nylon and linear low-density polyethylene, showed a reduced oxygen transmission rate compared with the same multilayer material lacking the protein layer [124]. A comparable effect was obtained by spreading the whey protein solution on a poly-lactic acid (PLA) film [125]. Performance measurements showed a 90% improvement in the oxygen barrier and 27% improvement in the water vapor barrier. This latter effect was achieved by adding either pectin (0.2%, *w*/*w*) or a commercial clay (Cloisite-30B) (0.5%, *w*/*w*) to the protein solution. Table 4 shows the water vapor permeability and mechanical properties (expressed as TS, and EAB) of casein and whey protein films. We can infer from these data that formulations of milk-protein-based films, with an appropriate amount of plasticizer, also offer a good barrier to water vapor diffusion through the thickness, without significantly compromising the mechanical performances of the material. A new milestone in the development of whey protein usage has been achieved, demonstrating the biodegradability of the whey coatings using enzymatic detergents [123]. In this context, the application of whey proteins and, more generally, milk-protein-based coatings in multilayer structures opens the door to a new generation of recyclable materials. A whey-protein-based layer could facilitate the separation of conventional petroleum-based plastics.

**Table 4:** Selected parameters of milk-protein-based films and coatings.

Type of Material	Formulation	Thickness	WVP	TS	EAB	Reference
Casein film		µm	(10^10^ g H_2_O Pa^−1^ s^−1^ m^−1^) (ASTM Method E96-95)	MPa (ASTM D1708-93)	% (ASTM D1708-93)	[81]
	NaCas 2.5% *w*/*v*;	110	5.4	57.0	4.0	
	NaCas 2.5% *w*/*v* + linseed oil resin/protein weight ratios: 0.05/0.1/0.15/0.2;	110	2.2–5.0	23.4–41.2	2.7–4.7	
	NaCas 2.5% *w*/*v* + Gly;	110	/	13.6	11.6	
	NaCas 2.5% *w*/*v* + tung oil.	110	/	25.8	3.0	
Casein film		µm	(10^10^ g H_2_O Pa^−1^ s^−1^ m^−1^) (ASTM E96M-10)	MPa (23°C, 50%RH)	% (23°C, 50%RH)	[71]
	Casein 10% *w*/*w*; Gly 50% *w*/*w* on protein;	800	11.0	2.6	458	
	Casein 10% *w*/*w*; Gly 50% *w*/*w* on protein; Tannic acid 4/8/10/15/20% *w*/*w* on protein.	800	4.6–5.7	2.6–5.8	294–458	
Active WP film		µm	(10^10^ g H_2_O Pa^−1^ s^−1^ m^−1^) (ASTM E96M-80)	MPa (ASTM D882-91)	% (ASTM D882-91)	[95]
	WPI 5% *w*/*v*; Sodium TPP 2 mM; Gly 1.8% *w*/*v*;	149	2.1	5.4	19	
	WPI 5% *w*/*v*; Sodium TPP 2 mM; Gly 1.8% *w*/*v*; WSCh 1.5/3% *w*/*w* on protein.	139–141	1.9–2.3	3.9–5.0	7–13	
WP film reinforced with CNCs				MPa (ASTM D882)	% (ASTM D882)	[84]
	WPI 5% *w*/*w*; Gly 50% dry matter;	/	/	2.30	46.07	
	WPI 5% *w*/*w*; Gly 50% dry matter; CNC (from 0% to 8% *w*/*w*).	/	/	3.41–4.93	17.63–26.54	
Antimicrobial casein-based flexible coating	Casein 10.9 g + TEA 2.6 g + 86 mL water; Caprolactam sol. 25% added drop-wise; ZnO NPs;	/	/	0.49	55.23	[113]
	ZnO NPs 0.1/0.5/1.0/2.0%.	/	/	0.97–1.65	52.51–64.77	
Casein-based TiO_2_ nanocomposite coating	Casein 15.7 g + TEA 3.9 g + 80 mL water; Caprolactam sol. 25% added drop-wise; Selected additives;	/	/	2.30	67.5	[114]
	Nano-TiO_2_ 0.0/0.1/0.2/0.3/0.4 g.	/	/	0.55–1.25	102–120	

WVP—water vapor permeability; TS—tensile strength; EAB—elongation at break; /= not determined.

## 5. Conclusions

The purpose of this review was to present the current status of research on food packaging materials based on milk proteins. This use of milk proteins is certainly of great interest to the food and the packaging industry because they can be used as “edible” materials, but in a broader and more modern sense, they can also improve and refine the technical and functional performances of conventional packaging materials. In fact, the current demand for food packaging is shifting towards the development of sustainable materials from renewable sources with high barrier properties and good processability. These materials can be used in packaging lines and to protect the quality of the most sensitive foods during their shelf life. Although great progress has been made on this topic, most findings remain at the laboratory stage. Milk proteins appear to be suitable for use in existing techniques as the main component in material formulations to produce both films and coatings. However, other aspects still hinder the upscaling of the proposed experimental materials to industrial application. The preparation of the film-forming matrices currently requires the usage of various additives, whose safety and suitability for food contact should be fully assessed. Issues such as long-term stability or the migration behavior of those substances into food have only been partly addressed thus far.

Nevertheless, this review evaluates recent advances in food preservation and food packaging with novel materials obtained from milk proteins, exploring the advantages and disadvantages of this approach. Directly comparing characteristics and technological performances of materials developed in different studies is very difficult since they are often evaluated under different conditions or reported using different units. However, gas barrier, antioxidant, antibacterial, mechanical, and water resistance properties, as well as the efficacy in the preservation of food items, were fully explored in every single study. The modification of milk protein structure using various approaches, such as crosslinking, either chemical or enzymatic, and physical treatments based on unconventional technologies, appears as a promising strategy for empowering their functional properties.

Overall, the suitability of milk proteins as biomaterials for composite film production seems to be consolidated. The commercial application of milk-protein-based materials in food packaging still requires studies that evaluate crucial aspects such as their sustainability and behavior and the long-term stability of additives included in packaged food items.

## Figures and Tables

**Table 1:** Relevant properties of milk proteins.

	α_s1_-Casein	α_s2_-Casein	β-Casein	κ-Casein	α-La	β-Lg
Molecular mass (Da)	23,614	25,230	23,983	19,023	14,174	18,362
Concentration g L^−1^	10	2.6	9.3	3.3	1.2	3.2
Pro	17	10	35	20	2	8
Cys	0	2	0	2	8	5
Glu	24	25	18	13	8	16
Asp	8	11	5	1	9	11
SerP	8	11	5	1	0	0
Glucidic residues	0	0	0	0–5	0	0
Hydrophobicity (kJ/res)	4.9	4.7	5.6	5.1	4.7	5.1
Isoelectric pH	4.16–4.49	4.68–5.13	4.50–5.29	5.43–6.12	4.66–4.90	4.64–4.98
Net charge/residue	−0.10	−0.07	−0.06	−0.02	−0.02	−0.04

Abbreviations: Pro (proline), Cys (cysteine), Glu (glutamic acid), Asp (aspartic acid), SerP (phosphoserine).

## Data Availability

Not applicable.
